# Dimensionality Reduction and Prediction of Impedance Data of Biointerface

**DOI:** 10.3390/s22114191

**Published:** 2022-05-31

**Authors:** Ebrahim Ismaiel, Anita Zátonyi, Zoltán Fekete

**Affiliations:** Research Group for Implantable Microsystems, Faculty of Information Technology & Bionics, Pázmány Péter Catholic University, H-1083 Budapest, Hungary; ismaiel.ebrahim@itk.ppke.hu (E.I.); zatonyi.anita@itk.ppke.hu (A.Z.)

**Keywords:** impedance analysis, neural interface, long short-term memory (LSTM) network, data prediction, data reduction

## Abstract

Electrochemical impedance spectroscopy (EIS) is the golden tool for many emerging biomedical applications that describes the behavior, stability, and long-term durability of physical interfaces in a specific range of frequency. Impedance measurements of any biointerface during in vivo and clinical applications could be used for assessing long-term biopotential measurements and diagnostic purposes. In this paper, a novel approach to predicting impedance behavior is presented and consists of a dimensional reduction procedure by converting EIS data over many days of an experiment into a one-dimensional sequence of values using a novel formula called day factor (DF) and then using a long short-term memory (LSTM) network to predict the future behavior of the DF. Three neural interfaces of different material compositions with long-term in vitro aging tests were used to validate the proposed approach. The results showed good accuracy in predicting the quantitative change in the impedance behavior (i.e., higher than 75%), in addition to good prediction of the similarity between the actual and the predicted DF signals, which expresses the impedance fluctuations among soaking days. The DF approach showed a lower computational time and algorithmic complexity compared with principal component analysis (PCA) and provided the ability to involve or emphasize several important frequencies or impedance range in a more flexible way.

## 1. Introduction

The biointerface is a physical contact area between a biological medium, including biological tissue, and a medium of another biomaterial component including organic/inorganic compounds [1]. The primary goal of biointerface technology is the understanding of interactions between biomolecules and corresponding surfaces or mediums, which has recently been considered significant in the fields of biology, biotechnology, diagnostics, and medicine [2]. Biointerface technology has widespread applications, for example, but not limited to, neural interfaces that deal with the nervous system for recording and stimulating purposes [3], pathogenesis and pathogen detection [4], membrane-based biosensing [5], nanotube/nanoparticle interfaces and cells in engineered microenvironments, and regenerative medicine [6].

Some biointerfaces consist of conductive parts and interaction with an electrical medium for recording, measuring, and/or stimulating purposes [7]. To measure the efficiency of the electrical performance of any device, we considered the impedance value, which is the measure of the opposition to the electrical current through the interface. Impedance measurement has various utilizations, such as examining the performance of the interface for long-term applications, biocompatibility with living tissue, and measuring the electrical impedance of biological tissues. In impedance sensing/measuring, a low amplitude of the variable frequency voltage signal is applied within the medium, and then the impedance is calculated based on the measured signal [8]. Electrochemical impedance spectroscopy (EIS) is an effective technique in the field of intermedium property analysis related to the interaction and response between the interface and the biomedium such as antibody recognition, cell capture, or response to surrounding electrical signals. Thus, EIS has been used in many important biomedical diagnostics and applications [9].

Therapies based on regenerative techniques are promising and emerging technologies that aim to produce stem cells and their progeny on a large scale. Concerning these techniques, impedance-based cellular assays (IBCAs) are considered a sufficient approach to study stem cell properties; furthermore, they can deliver quantitative and highly sensitive results that can be easily automated and scalable, and the literature has demonstrated its ability to monitor stem cell regeneration, differentiation, and maturation [10,11]. Transdermal drug delivery and biomarker sampling applications are strongly influenced by skin hydration dynamics, where skin permeability proportionately increases with higher hydration. EIS is a very proper technique to study and evaluate the hydration process, because the changes in the electrical properties of the stratum corneum (SC) are strongly influenced by the hydration state [12]. Neural implants manufacturing and testing technologies has become one of the most important fields in the neuroscience domain, especially in neural interface and prosthetic systems that could be used for diagnosis, monitoring, compensation for affected neural systems and, later, to provide treatment for neurological diseases or to stimulate functional domains [8]. The ideal electrode for neural interfaces is described as a tool that allows signals to be recorded precisely and with the lowest level of its own influence. To ensure the long-term reliability of the neural implants, it is important to examine the major modes of failure during long-term implantation by conducting pre-in vitro experiments using EIS. While the foreign body or medium biocompatibility with the neural probe or microarray is the most influential factor on the neural interface, there are other transient states of failure that affect the extracellular recordings negatively [13]. Hence, impedance analysis circuits during in vivo and clinical applications have been implemented in many neural interfaces to observe and examine chronically the efficiency of recording/stimulating devices [14,15]. Long-term impedance analysis is also used to examine feedback-based electrode rehydration for long-term biopotential measurements [16].

Based on the above, EIS performance and impedance analysis are the golden tools for many emerging biomedical applications. In contrast, those types of measurements have multidimensional outcomes related to the environment, frequencies, and applied voltage. Thus, they require huge efforts to track or design online diagnostic systems, which will rely on machine learning tools in the near future. Dimensionality reduction of impedance data has been reported previously in several in vivo applications. Impedance indices are proposed to extract pertinent features from the impedance spectrum, such as the magnitude index (MIX), the phase index (PIX), the real part index (RIX), and the imaginary part index (IMIX), where those indices compare values at 20 and 500 kHz to diagnose oral mucosa [17]. Known approaches of dimensional reduction have been applied to bioimpedance readings of two plant-based biological materials (i.e., Solanum tuberosum L. and Malus domestica). The implemented approaches were principal component analysis (PCA), locally linear embedding (LLE), metric multidimensional scaling (mMDS), and isomaps, where PCA was approved as the most suitable one [18]. The efficiency of reduction using PCA was validated by implementing a classification approach of EIS features based on a multilayer perceptron (MLP) neural network, and the accuracy of the classification before and after utilizing PCA was the same [18]. Some relevant work on predicting impedance in various applications showed a good ability to estimate impedance changes based on available data. A convolutional neural network (CNN) was used in predicting impedance spectra over battery life in the fully charged and fully discharged states, and the results proved the ability of CNN to monitor battery performance based on impedance data [19]. Radial basis function neural network (RBFNN) was also used to predict impedance behavior based on the Nyquist plot, and the results showed a good similarity with actual values in different situations of crude oil in transport industry environments [20]. Long short-term memory (LSTM) was utilized as a prediction tool to measure the degradation of a fuel cell (FC) stack using EIS data, and the reported results of prediction were close to the actual behavior [21].

The related works (Table 1) show that data reduction or feature extraction is a promising approach for experts to deal with a small amount of data with the same efficiency and accuracy of the content [17,18]. On the other hand, the ability of ML tools in predicting the behavior of a physical/biointerface that contains electrochemical measurements has been proved [19,20,21]. This paper presents an approach for the dimensional reduction of big impedance data, called day factor (DF), which converts EIS data over many days of an experiment into a one-dimensional sequence of values. The DF aims to provide a flexible formula with low computational time that can combine the purposes in [17,18] and provide simpler input for ML [19,20,21]. Then, we used the LSTM network to predict the future behavior of the DF, which gives specialists potential knowledge regarding upcoming changes in impedance. Finally, we illustrate and discuss the ability of the proposed approach to predict each next day of soaking days depending on the previous days, in addition to discussing DF features compared with PCA.

## 2. Materials and Methods

### 2.1. ECoG Microarrays

The impedance dataset of this study was established based on the characterization of the recording sites of three neural microarrays using EIS. The properties of each microarray are illustrated as follows.

#### 2.1.1. Polyimide-Based ECoG Microarray: “Microarray 1”

**Figure 1:**
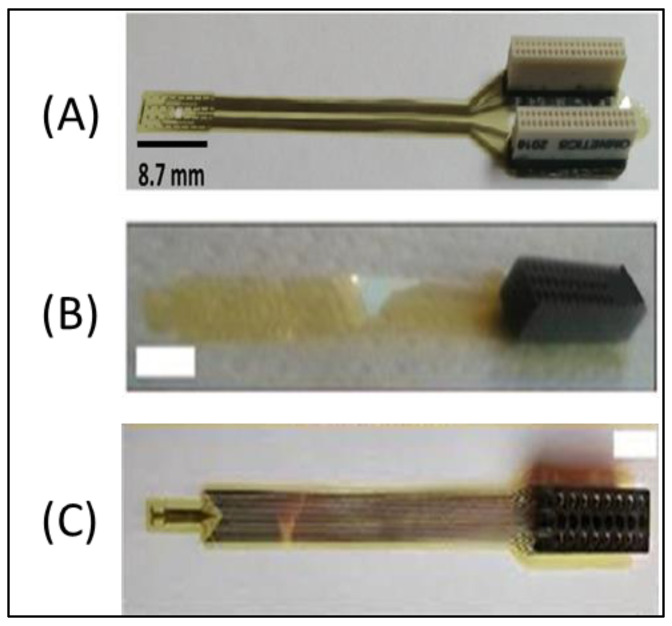
Tested ECoG microarrays: (**A**) Microarray 1; (**B**) Microarray 2; (**C**) Microarray 3.

The flexible micro-ECoG device (Figure 1A) was fabricated at the at Institut für Mikrosystemtechnik, University of Freiburg, Germany, with the following properties and dimensions: thickness equal to 10 µm; 150 mm (6′′) of silicon wafers; 5 µm thick polyimide (UBE UPIA-ST1001, UBE Industries Ltd., Tokyo, Japan) was spin-coated and annealed; AZ9260 and AZ5214E (Microchemicals GmbH, Ulm, Germany) photoresist sites formed the lift-off pattern; a platinum conductive layer equal to 250 nm was sandwiched between two layers of 25 nm titanium. Thirty-two recording sites with a 4 × 8 matrix configuration was used to record the ECoG signals. This device has shown outstanding performances in in vivo and in vitro applications [22].

#### 2.1.2. Polyimide-Based ECoG Microarray: “Microarray 2”

A 32-channel microelectrode array (Figure 1B) was formed using a polyimide (PI) substrate and indium–tin–oxide (ITO) metallization eight microns thick. Polyimide (PI2611, HD Microsystems GmbH, Heidelberg, Germany) was spin-coated on a 4” silicon wafer. Indium–tin–oxide was deposited in RF sputtering equipment. A second layer of polyimide, as a top passivation layer, was spin coated at 6000 rpm for 45 s. One hundred nanometers of aluminum thin film, as a hard mask for RIE etching, was deposited in e-beam evaporator equipment. The aluminum layer was selectively opened above the recording sites and electrode contour by aluminum etchant through a photoresist mask patterned by photolithography. Pads in the backbone of the electrode were mounted on a 2 × 16 channel PreciDiP electrical connector using silver epoxy glue baked at 100 °C [23].

#### 2.1.3. Polyimide-Based ECoG Microarray: “Microarray 3”

A polyimide-based flexible ECoG microarray (Figure 1C) with 16 electroplated platinum recording sites, eight microns thick, was fabricated in the Microsystems Laboratory of the Centre for Energy Research [8].

### 2.2. Characterization of ECoG Microarrays Using EIS

The three mentioned ECoG microarrays in this paper were characterized using the EIS tool for 11 soaking days with different parameters and instruments as shown in Table 2. The impedance amplitude and phase values of each frequency/soaking day were preprocessed and saved in a compatible format using MATLAB.

### 2.3. Dimensionality Reduction of Impedance Data

Bode and Nyquist diagrams are the most common data representation tools of impedance measurements that give abstract information about the electrical performance of the tested interface [9], but they need individual tracking and interpretation regarding the range of tested frequencies and soaking days.

To avoid this complexity in EIS evaluation and to prepare preprocessed data for the prediction of neural interface impedance and to provide some flexibility in features contribution, we propose and implemented a new formula that converts multidimensional EIS outcomes (impedance amplitude/frequency/soaking day) to a one-dimensional signal or series of values according to EIS days as shown in Equations (1) and (2):

(1) $${\mathrm{DF}}_{d}=\frac{\sum_{k=1}^{f_{end}}\left(\left(\frac{1}{f_{k}}\right)*\left|log\left(Z_{k}\right)\right|\right)}{DF_{1}}$$

(2) $${\mathrm{DF}}_{1}=\overset{f_{end}}{\underset{k=1}{\sum }}\left(\left(\frac{1}{f_{k}}\right)*\left|log\left(Z_{k}\right)\right|\right)$$

where:

- ${\mathrm{DF}}_{d}$: Day factor of (*d*) day, or normalized DF of (*d*) day to the first day ${\mathrm{DF}}_{1}$;
- ${\mathrm{DF}}_{1}$: Day factor value of the first day;
- $\frac{1}{f_{k}}\;$: Proportional values of testing frequency;
- $Z_{k}$: Impedance value at $\;f_{k\;}$ frequency;
- $\left|log\left(Z_{k}\right)\right|\;$: Logarithm values of impedance amplitude at $f_{k\;}$ frequency in day (*d*).

The name of the formula is day factor (DF) which expresses the EIS measurements during the same day in the normalized form to the day factor of the first soaking day. The unique value of ${\mathrm{DF}}_{d}$ (for EIS of the day {*d*}) was normalized to the ${\mathrm{DF}}_{1}$ to visualize how far or close the EIS outcomes became to the EIS of the first day; in other words, we considered that the EIS of the first day was the reference and optimal value of the interface impedance, and any change in it would affect the signal shape of DF. This values series of DF(s) represents proportional values to the first-day factor and only gives a relative overview of the interface behavior. A more detailed interpretation of the DF formula and its correlation with the EIS impedance spectrum is demonstrated in the Section 3.

Since DF values represent only soaking days, the outcomes will be discrete values, and soaking days can have a time gap of at least two or three days between each sequenced soaking day. Therefore, to convert the DF values into a continuous signal, we used the Curve Fitting Toolbox™ functions in MATLAB, which allowed us to fit to and smooth the DF data points.

### 2.4. Long Short-Term Memory (LSTM) Network

After preparing a one-dimensional DF signal of values that expressed the impedance behavior of the neural interface. We used the principle of time series forecasting or signal behavior prediction using an LSTM network.

An LSTM network is a type of recurrent neural that uses a series of observations to become familiar with sequence prediction problems. LSTM is specifically designed to avoid the long-term dependency problem and save/retrieve information for long periods. By training a sequence-to-sequence regression after shifting the training data for one-time step, we would be able to predict the values of the next time steps for the same data sequence [24].

The LSTM network in this research was used as a forecasting method [25] for a values sequence or signal by training the neural network on DF values and checking the difference between the predicted behavior and the actual one. The principle is easy: at each time step of the input sequence, the LSTM network learns to predict the value of the next time step. Because the time step is used for prediction, which is the soaking day, we see a one-day step time shift in the prediction sequence. An LSTM network was created with 200 hidden units, and the solver was “adam” and trained for 250 epochs. The gradient threshold was set to 1. The initial learning rate was 0.005. The network was trained using ${\mathrm{DF}}_{1\to \left(n-1\right)}$ data, then it predicted ${\mathrm{DF}}_{n}$, where (n) is the predicted DF part or the upcoming soaking day. The LSTMs in this paper were implemented using the Deep Learning Toolbox (MathWorks, Natick, MA, United States) [26]. All computations were performed on a laptop that had a CPU Intel(R) CoreI i5-4300U CPU @ 1.90 GHz, 2494 MHz, 2 core(s), and 4 logical processor(s).

### 2.5. Performance Evaluation of Prediction

The fitted DF signal after implementing splines approximation became the high-sampled signal. By predicting each DF part using LSTM, we would had actual and predicted signals. The traditional metrics for measuring performance, such as accuracy and sensitivity, were not useful for evaluating the outcome of our prediction approach. To obtain more informative measures, we propose two ways to evaluate prediction accuracy as follows.

#### 2.5.1. Accuracy of Final State (AFS)

To measure the accuracy of predicted DF as an absolute or quantitative value, we calculated the difference between the last value of the actual DF signal and the last value of the predicted one. Then, we normalized it to the maximum range of the actual DF, which gave us the relative error of the predicted signal; thus, (1-error) expresses the accuracy of the equality between the actual and the predicted DF as shown in Equation (3).

(3) $$AFS\left(\%\right)=\left(1-\frac{Value_{predict}-Value_{actaul}}{Max\left(DF_{actaul}\right)-Min\left(DF_{actaul}\right)}\right)*100$$

#### 2.5.2. Accuracy of Correlation Coefficients (ACCs)

After measuring the final value quantitatively, we should evaluate the behavior of predicted signals such as the slope and the similarity with the actual one. Where the correlation coefficient of two random variables is a measure of their linear dependence, we can thus consider the correlation coefficient of the two actual and predicted DF as an accurate measure of similarity. If each DF signal has N scalar observations, then the Pearson correlation coefficient, in percentage, is defined as in Equation (4):

(4) $$\mathrm{ACC}\left(\%\right)=\left|\frac{cov\left(A,B\right)}{\sigma_{A}\sigma_{B}}\right|*100$$

where:

- ***cov* (*A*,*B*)**: The covariance between two random variables *A* and *B*;
- $\sigma_{A}$: Standard deviation of *A*.

## 3. Results

The Results Section handles each microarray separately by collecting the impedance data of the examined microarray, implementing the day factor (DF) formula on the impedance data and, finally, using the DF signal with the LSTM network to examine the impedance prediction of each post-soaking day based on the previous days as training input. The used ECoG microarrays contained many recording sites that could be characterized; thus, the upcoming results are concerned with the average impedance amplitude of all recording sites, which are expressed as one impedance value for each frequency and soaking day.

### 3.1. Day Factor Prediction of the Microarray 1 Impedance Data

**Figure 2:**
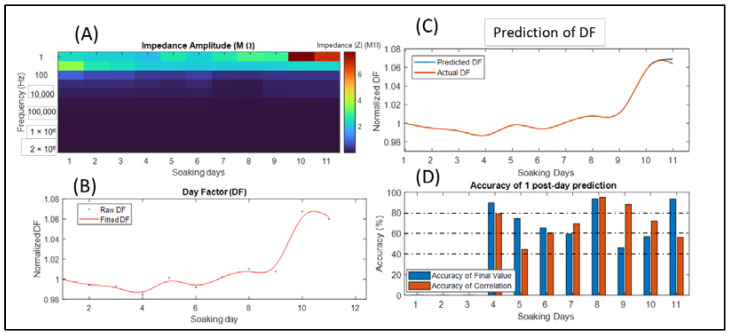
Reduction and prediction of the impedance data in Microarray 1: (**A**) impedance values according to frequencies and soaking days; (**B**) approximated DF signal using impedance in each soaking day, where the blue dots refer to the original DF values and the brown line to the fitted curve using splines; (**C**) the actual DF with the predicted day 11 using the LSTM shown as the blue line; (**D**) accuracy of the final state AFS (brown) and the accuracy of the correlation coefficient ACC (blue) of each predicted soaking day starting from the fourth day.

The impedance analysis of the 11 soaking days showed stable values of impedance as shown in Figure 2A, where the impedance ranged between 100 and 200 kΩ at 1 kHz. The last two days showed higher impedance, especially at 1 Hz.

By implementing the DF formula using Equations (1) and (2), we obtained the DF signal (Figure 2B) that expressed the behavior of all frequencies during soaking days. The DF, or normalized DF, depicts the changes in the impedance by normalizing the DF of each soaking day to the DF of the first day. On day 6 (Figure 2A), we can notice a slight reduction in the impedance, where this change clearly occurred in the DF (Figure 2B). During the last two days, the impedance became higher, which affected the DF signal by increasing it too (Figure 2A,B).

Starting from the third soaking day, we considered the DF values of the previous days as training data for the LSTM network; then, we used the LSTM to predict the next day, which was the fourth day (for example). We repeated the procedure by predicting the fifth day using the last four days, etc. The predicted and actual DF data of day 11 are visualized in Figure 2C, where we can see that the predicted values were relatively close to the actual ones, but the slope and steps were different, which made the accuracy of the final value (AFS) higher than 90% with a low accuracy of the correlation coefficient (ACC), respectively (Figure 2C,D).

The prediction accuracy for each soaking day using the previous days is illustrated in Figure 2D, where we can notice that the AFS (error percentage of the predicted value to the total behavior) was generally above 60%, and during slight changes, it was able to predict the exact values for the next day. On the other hand, the ACC (similarity between the actual and the predicted DF behavior) showed a high value during the lower values of the final value accuracy, which proved that while the LSTM could not reach the next actual value, it could predict the same slope and behavior for the next day.

### 3.2. Day factor Prediction of the Microarray 2 Impedance Data

**Figure 3:**
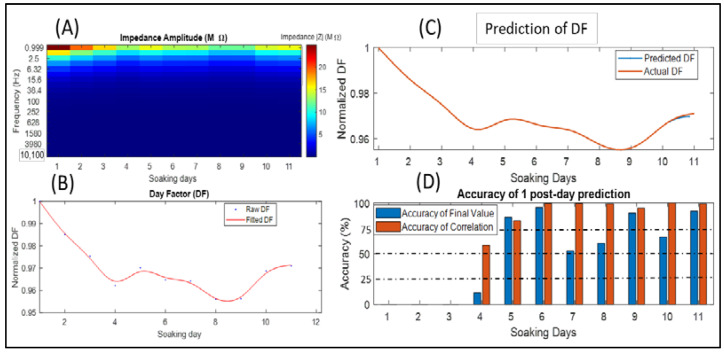
Reduction and prediction of the impedance data in Microarray 2: (**A**) impedance value according to frequencies and soaking days; (**B**) approximated DF signal using impedance in each soaking day, where blue dots refer to the original DF values and the brown line to the fitted curve using splines; (**C**) actual DF with the predicted day 11 using the LSTM shown as the blue line; (**D**) accuracy of the final state AFS (brown) and the accuracy of the correlation coefficient ACC (blue) of each predicted soaking day starting from the fourth day.

The impedance measurements of the 11 soaking days are depicted in Figure 3A, where the impedance ranged between 50 and 250 kΩ at 1 kHz. By implementing the DF formula using Equations (1) and (2), we obtained the DF signal (Figure 3B), where we can notice a slight reduction in the impedance, while this change clearly occurred in the DF (Figure 3A). During the last two days, the impedance increased, which affected the DF signal by increasing it too (Figure 3A,B).

The predicted values of day 11 were relatively close to the actual one, in addition to the slope and steps, which made the accuracy of the AFS and ACC higher than 90% (Figure 3C,D).

The prediction accuracy for each soaking day using the previous days is illustrated in Figure 3D, where we can notice that the accuracy of the final value mostly showed good accuracy (above 75%), and during slight changes, it was able to predict exact values for the next day.

### 3.3. Day Factor Prediction of the Microarray 3 Impedance Data

**Figure 4:**
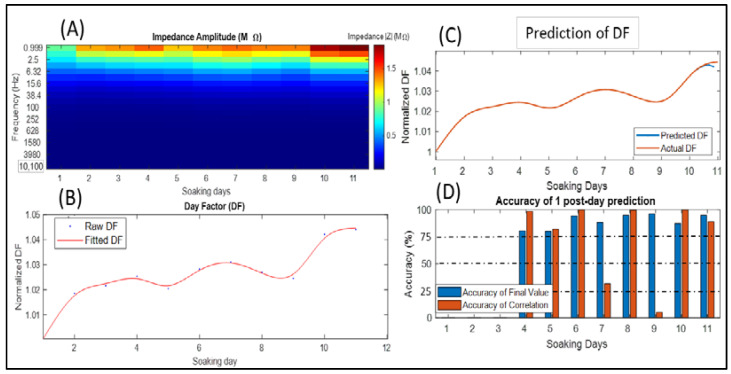
Reduction and prediction of the impedance data in Microarray 3: (**A**) impedance value according to frequencies and soaking days; (**B**) approximated DF signal using the impedance in each soaking day, where the blue dots refer to the original DF values and the brown line to the fitted curve using splines; (**C**) actual DF with the predicted day 11 using the LSTM shown as the blue line; (**D**) accuracy of the final state AFS (brown) and the accuracy of the correlation coefficient ACC (blue) of each predicted soaking day starting from the fourth day.

The impedance analysis of the 11 soaking days showed stable values of impedance as shown in Figure 4A, where the impedance ranged between 70 and 210 kΩ at 1 kHz.

The normalized DF depicts the changes in the impedance, where on day 5 and 9 during soaking (Figure 4A), we can notice a slight reduction in the impedance, while this change clearly occurred in the DF (Figure 4B). The impedance increased during the last two days, which affected the DF signal by increasing it too (Figure 4A,B).

The predicted and actual DF data of day 11 are visualized in Figure 4C, where the AFS and ACC showed an accuracy higher than 90% (Figure 4C,D).

The prediction accuracy of each soaking day using the previous days is illustrated in Figure 4D and mostly showed good accuracy (i.e., above 75%). Even during slight changes, it could predict the exact values for the next day. On the other hand, the ACC showed a high value during the lower AFS, except for day 7 and 9, where the LSTM was not able to provide a good slope for the predicted values.

### 3.4. Comparison between the DF and PCA Approach

**Figure 5:**
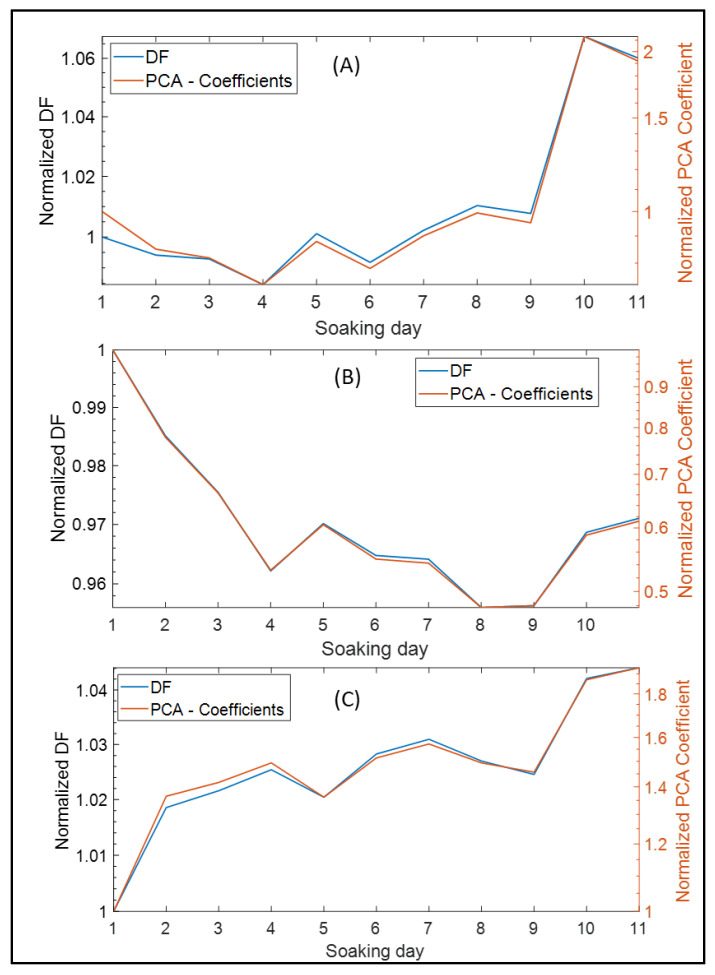
Implementing DF and PCA on the EIS dataset of the three microarrays: (**A**) normalized DF and PCA using the Microarray 1 impedance data; (**B**) normalized DF and PCA using the Microarray 2 impedance data; (**C**) normalized DF and PCA using the Microarray 3 impedance data.

PCA is a very popular dimensionality reduction technique. PCA has been applied in many fields of bioinformatics [27] and, especially, in data reduction of EIS outcomes [18]. The main aim of manipulating impedance data is the limitation of hardware and software capabilities in embedded systems [18]. In this section, we compare the results of DF with PCA for each neural interface. PCA was implemented in MATLAB, where the PCA centered the data and used the singular value decomposition (SVD) algorithm and returned one component only as a result. The PCA used the same dataset of the EIS that consisted of the mean impedance for all channels in terms of frequency and soaking days, and the resulting coefficients of the PCA were visualized after normalizing all coefficients to the first coefficient as shown in Figure 5. The similarity between DF and PCA for each EIS datum (Figure 5) is clear with different scale or quantitative values, which increases the creditability of the DF approach for reducing EIS data. On the other hand, by comparing the computational time for implementing or executing DF and PCA (Table 3), the required time for executing the PCA was 17 times higher than for DF, and this meets the main objectives of data reduction in EIS systems [18].

## 4. Discussion

Biointerface technology has become an important addition to many medical and clinical applications. Its main use as a biomedium tester or monitor requires long-term reliability and periodic evaluation of its performance in terms of its ability to obtain accurate measurements from targeted areas. EIS has been used widely in measuring the impedance and electrochemical properties of any biointerface device, where EIS can provide information regarding the interface and surrounding environment.

Concerning neural interface, EIS measurements can provide a detailed examination regarding the electrical properties and the biostability of neural implants and microarrays either in in vitro or in vivo applications. Recently, measuring circuits of EIS were embedded within the structure of neural microchips to conduct periodic EIS tests and to evaluate the efficiency of long-term implantation in living tissue [15]. In the same context, regardless of the biointerface type or application, the EIS procedure results in large quantities of data in terms of frequency, trails, and channels. Experts need to track changes in EIS outcomes and decide whether the impedance behavior for each channel and frequency is moving within a normal range or if an interface failure is starting to emerge. In this research, we proposed an approach that can summarize the multidimensional data of EIS and convert them into a one-dimensional signal that expresses the overall changes in the impedance in a relative way by normalizing EIS trails to the early first trail. This research addressed a case study of three types of neural interfaces. Impedance measurements were conducted based on the purposes of in vitro characterization. In this study, the most important values of frequency were between 1 Hz and 10 kHz, which are the standard and approved range of testing frequencies. Moreover, the lower frequencies in our approach had more of an effect compared with the higher ones. The impedance data for “Microarray 1” had EIS using frequencies of 1 Hz to 2 MHz, whereas other microarrays were characterized with 0.1 Hz to 10 kHz and that was achieved to examine the DF outcomes with different parameters. All used impedance data were collected based on EIS measurements of specific types of neural interfaces with fixed and limited values of RMS and frequency (Table 2). Our proposed approach for impedance data reduction achieved the desired purpose of summarizing the impedance behavior into one abstract and normalized signal, called normalized day factor, as shown in Figure 2B, Figure 3B and Figure 4B. Normalizing each DF for each soaking day to the DF of the early first soaking day as a concept gives more informative behavior of the DF, because, apparently, it reflects the change into better (i.e., lower than 1) and worse (i.e., higher than 1) one-dimensional shifts and gives the reader a fast impression regarding the total behavior of the impedance over all soaking days. In the DF and impedance graphs of Microarray 1 (Figure 2A,B), the fluctuating and slight changes in the impedance for all frequencies between day 4 and 6 appeared clearly in the DF signal, and the steep increase during the last two days (e.g., day 10 and 11) of the DF was related to the high impedance increasing at all frequencies. Concerning the Microarray 2 and Microarray 3 devices, the DF signals (Figure 3B and Figure 4B) represent visibly the total changes in the impedance values at all frequencies and soaking days (Figure 3A and Figure 4A), even with slight changes in the impedance value.

By comparing the performance of DF and PCA using the same datasets, we found that both give the same distribution and resolution of impedance changes, but DF had two better features than PCA. The first feature is the lower computational time and algorithmic complexity (Table 3). This feature is useful in circuit modeling of the electrical properties of biological tissues [18] and can provide faster data reduction during the real-time execution of electrical impedance tomography [28], which demands microchips with faster computational approaches. We should mention that our research did not provide an implementation of DF and PCA in microchips, and because of that, our conclusions were based on PC-based implementation (Table 3). Those small differences in computational time between DF and PCA, such as 4.1 msec and 74.5 msec, can have much influence on the response of microchips compared with PC, which has higher processing capabilities. Despite there being no related work on the exact comparison, we hypothesize that in the near future the development of microsystems is going to consider faster and more accurate data reduction [18] within microchips [15]. The second feature is that the DF formula can be modified easily and provide the ability to involve or modulate some important frequencies or impedance range, which meets the main objectives of data reduction in EIS systems [18].

The DF signal or the proposed approach for the impedance data reduction can offer or suggest a solution for clinical and biomedical applications. For example, localized bioimpedance analysis (BIA) can assess soft tissue hydration and cell membrane integrity using noninvasive electrical measurements, but the main challenge in this technique is to standardize a protocol that allows for the assessment of any person [29], where DF can ease the solution in the same context by concentrating on the most mutual range of changes among subjects and modulating the DF formula to achieve that. Another clinical application is electrical impedance tomography (EIT), which is an imaging method of the internal structure of the biological tissue based on the conductivity of different tissues inside the organism, where the accuracy of the measured data plays the main role in the development of image reconstruction [28]. By going back to the second feature of the DF, concerning manipulating the interesting frequencies and impedance values, the DF can provide a solution for reducing the total data collection time.

Today, LSTM applications are mainly concerned with predicting upcoming events or signals [25,30,31]. In our research, we used the LSTM network, and its main use was as a forecasting tool of a signal [30,31] to study the ability to estimate upcoming changes in impedance behavior using the normalized DF signal which briefly describes the impedance changes. The prediction of DF is implemented using the DF values of previous soaking days as a training dataset and the next soaking day as a targeted value, and because we mentioned earlier that the DF signal is resampled to a larger number of samples, the targeted result or predicted DF is a series of values that represents the predicted soaking day. The AFS and ACC values of predicting the DF of Microarray 1 (Figure 2D) expressed the ability of the LSTM to estimate the upcoming changes in the DF, where we can see that the accuracy of the quantitative values (AFS) and their shape similarity to the actual DF change (ACC) reached 60%, which was not so unfavorable compared with the fluctuating changes in the DF. In contrast, a high accuracy larger than 70% occurred during normal behavior of the DF. On the other hand, the AFS and ACCs of predicting Microarray 2 and Microarray 3 (Figure 3D and Figure 4D) showed better performances compared with Microarray 1. In general, an LSTM uses the observations of each time sequence [24,30], and because the DF signal is mostly nonperiodic and does not contain repeated events with intervals, it is considered a challenge to predict its next state. To avoid the confusion of an LSTM observation, we can enhance the prediction by using fewer numbers of soaking days for training. However, even with low values for AFS, the LSTM was able to provide an approximate behavior of the DF as slope and direction and considered a good estimation of the DF’s changes. We considered both AFS and ACC with values between 75% and 100% as having good accuracy, because it refers to an acceptable prediction margin that keeps the orientation of the DF signal in the same correct behavior (Figure 2C, Figure 3C and Figure 4C). Where the DF aims to summarize the changes in the impedance at a specific range of frequencies, the LSTM with a minimum prediction accuracy of 75% can be considered as an alarm for the occurrence of high impedance at any frequency. The proposed method can provide a solution for the analysis of big impedance data in terms of cycles/time for many applications of impedance spectroscopy such as fuel cell degradation and diagnosing battery life [19,21]. Concerning related work, deep learning tools are used in many approaches that aim to predict the EIS data in many fields [19,20,21], and an LSTM was proposed previously to predict the degradation of EIS in a Nyquist diagram [21]. Compared with our approach, the prediction of a DF signal that summarizes the total EIS data gave more flexibility and informative predictions of EIS data.

## 5. Conclusions

In this research, we proposed an approach that can summarize the multidimensional data of EIS and convert them into a one-dimensional signal (DF) that expresses the overall changes in the impedance in a relative way by normalizing EIS trails to the early first trail. The DF approach showed a lower computational time and algorithmic complexity compared with PCA and provided the ability to easily involve or emphasize several important frequencies or impedance range. An LSTM network was used with the DF signal to study the ability to predict the potential changes that could be used soon within microchip structures to predict and avoid undesirable consequences. The DF formula could be generalized to express the phase changes in the impedance and use the DF of the amplitude and phase to obtain a more powerful estimation of the biointerface performance.

## Figures and Tables

**Table 1:** Comparison table of related works.

Reference	Dimensionality Reduction/Data/Features Extraction Tool	ML Technique for Prediction	Purpose
[17]	MIX, PIX, RIX, and IMIX	-	Diagnosing oral mucosa with a lower number of informative features.
[18]	PCA, LLE, mMDS, and Isomaps	MLP neural network	Dimensionality reduction of impedance data and keeping the important and informative content.
[19]	Constant current (CC) charging data	CNN	Predicting impedance spectra over a battery’s life.
[20]	Nyquist plot	RBFNN	Electrochemical impedance prediction in the presence of a corrosion inhibitor.
[21]	Cell voltage through cycles of FC usage	LSTM	Predicting the degradation of an FC stack.

**Table 2:** Properties of the EIS procedures for each microarray.

ECoG Microarray	Potentiostat	Medium	RMS ^1^	Soaking Days	Frequencies
Microarray 1	nanoZ ^2^	PBS ^4^	4 mV	11 days	1 Hz to 2 MHz
Microarray 2	Gamry ^3^	PBS	25 mV	11 days	0.1 Hz to 10 kHz
Microarray 3	Gamry	PBS	25 mV	11 days	0.1 Hz to 10 kHz

^1^ Root mean square of sinusoidal electric signal; ^2^ nanoZ Impedance Tester (Plexon, Texas); ^3^ Gamry Reference 600 Potentiostat (Gamry Instruments, Warminster, PA, USA); ^4^ 0.01 M phosphate-buffered saline (PBS) solution (P4417, tablet diluted in 200 mL distilled water, Merck KGaA, Darmstadt, Germany).

**Table 3:** Elapsed time in milliseconds to execute DF and PCA using MATLAB.

ECoG Microarray	DF	PCA
Microarray 1	4.1	74.5
Microarray 2	4.2	84.7
Microarray 3	3.9	67

## Data Availability

The datasets generated and/or analyzed during the current study are available from the corresponding author upon reasonable request.

## References

[1] Lesiak-Orłowska B. (2022). Surfaces and Interfaces in Biocatalysis. Catalysts.

[2] Vianello F., Cecconello A., Magro M. (2021). Toward the Specificity of Bare Nanomaterial Surfaces for Protein Corona Formation. Int. J. Mol. Sci..

[3] Guo B., Fan Y., Wang M., Cheng Y., Ji B., Chen Y., Wang G. (2021). Flexible Neural Probes with Electrochemical Modified Microelectrodes for Artifact-Free Optogenetic Applications. Int. J. Mol. Sci..

[4] Ghosh S., Lahiri D., Nag M., Dey A., Sarkar T., Pathak S.K., Atan Edinur H., Pati S., Ray R.R. (2021). Bacterial Biopolymer: Its Role in Pathogenesis to Effective Biomaterials. Polymers.

[5] Gayda G.Z., Demkiv O.M., Stasyuk N.Y., Serkiz R.Y., Lootsik M.D., Errachid A., Gonchar M.V., Nisnevitch M. (2019). Metallic Nanoparticles Obtained via “Green” Synthesis as a Platform for Biosensor Construction. Appl. Sci..

[6] Biru E.I., Necolau M.I., Zainea A., Iovu H. (2022). Graphene Oxide–Protein-Based Scaffolds for Tissue Engineering: Recent Advances and Applications. Polymers.

[7] Horváth Á.C., Borbély S., Boros Ö.C., Komáromi L., Koppa P., Barthó P., Fekete Z. (2020). Infrared neural stimulation and inhibition using an implantable silicon photonic microdevice. Microsyst. Nanoeng..

[8] Zátonyi A., Fedor F., Borhegyi Z., Fekete Z. (2018). In vitro and in vivo stability of black-platinum coatings on flexible, polymer microECoG arrays. J. Neural Eng..

[9] Magar H.S., Hassan R.Y.A., Mulchandani A. (2021). Electrochemical Impedance Spectroscopy (EIS): Principles, Construction, and Biosensing Applications. Sensors.

[10] Gamal W., Wu H., Underwood I., Jia J., Smith S., Bagnaninchi P.O. (2018). Impedance-based cellular assays for regenerative medicine. Phil. Trans. R. Soc..

[11] Morgan K., Gamal W., Samuel K., Morley S.D., Hayes P.C., Bagnaninchi P., Plevris J.N. (2020). Application of Impedance-Based Techniques in Hepatology Research. J. Clin. Med..

[12] Morin M., Ruzgas T., Svedenhag P., Anderson C.D., Ollmar S., Engblom J., Björklund S. (2020). Skin hydration dynamics investigated by electrical impedance techniques in vivo and in vitro. Sci. Rep..

[13] Fekete Z., Pongrácz A. (2017). Multifunctional soft implants to monitor and control neural activity in the central and peripheral nervous system: A review. Sen. Actu. B Chem..

[14] Munge A., Sankar V., Sendi M.S., Ghovanloo M., Guler U. A bio-impedance measurement IC for neural interface applications. Proceedings of the 2018 IEEE Biomedical Circuits and Systems Conference (BioCAS).

[15] Iwagami T., Tani T., Ito K., Nishino S., Harashima T., Kino H., Kiyoyama K., Tanaka T. Area-Efficient and Wide-Range Impedance Analysis Circuit for Multichannel High Quality Brain Signal Recording System. Proceedings of the 2015 International Conference on Solid State Devices and Materials.

[16] Krishnan A., Weigle H., Kelly S., Grover P. Feedback-based Electrode Rehydration for High Quality, Long Term, Noninvasive Biopotential Measurements and Current Delivery. Proceedings of the 2019 IEEE Biomedical Circuits and Systems Conference (BioCAS).

[17] Lackovi I., Stare Z., Protulipac T. (2003). On the applicability of electrical impedance indices to characterize the condition of the oral mucosa. WIT Trans. Biomed. Health.

[18] Cavalieri R., Bertemes-Filho P. Dimensionality reduction methods for Impedance Spectroscopy data of biological materials. Proceedings of the 4th Latin American Conference on Bioimpedance 2021 (CLABIO 2021).

[19] Duan Y., Tian J., Lu J., Wang C., Shen W., Xiong R. (2021). Deep neural network battery impedance spectra prediction by only using constant-current curve. Energy Storage Mater..

[20] Komijani H., Rezaeihassanabadi S., Parsaei M.R., Maleki S. (2017). Radial basis function neural network for electrochemical impedance prediction at presence of corrosion inhibitor. Peri. Polyt. Chem. Eng..

[21] Caponetto R., Guarnera N., Matera F., Privitera E., Xibilia M.G. Application of Electrochemical Impedance Spectroscopy for prediction of Fuel Cell degradation by LSTM neural networks. Proceedings of the 2021 29th Mediterranean Conference on Control and Automation (MED).

[22] Csernyus B., Szabó Á., Fiáth R., Zátonyi A., Lázár C., Pongrácz A., Fekete Z. (2021). A multimodal, implantable sensor array and measurement system to investigate the suppression of focal epileptic seizure using hypothermia. J. Neural. Eng..

[23] Zátonyi A., Borhegyi Z., Cserpán D., Somogyvári Z., Srivastava M., Kisvárday Z., Fekete Z. (2017). Optical Imaging of Intrinsic Neural Signals and Simultaneous MicroECoG Recording Using Polyimide Implants. Proceedings.

[24] Wang J., Jiang W., Li Z., Lu Y. (2021). A New Multi-Scale Sliding Window LSTM Framework (MSSW-LSTM): A Case Study for GNSS Time-Series Prediction. Remote Sens..

[25] Cai C., Tao Y., Zhu T., Deng Z. (2021). Short-Term Load Forecasting Based on Deep Learning Bidirectional LSTM Neural Network. Appl. Sci..

[26] The MathWorks I. (2022). Deep Learning Toolbox. https://www.mathworks.com/help/deeplearning/index.html.

[27] Scholz M. (2006). Approaches to Analyse and Interpret Biological Profile Data. Doctoral dissertation.

[28] Shi Y., Yang Z., Xie F., Ren S., Xu S. (2021). The Research Progress of Electrical Impedance Tomography for Lung Monitoring. Front. Bioeng. Biotechnol..

[29] Cebrián-Ponce Á., Irurtia A., Carrasco-Marginet M., Saco-Ledo G., Girabent-Farrés M., Castizo-Olier J. (2021). Electrical impedance myography in health and physical exercise: A systematic review and future perspectives. Front. Physiol..

[30] Kolaghassi R., Al-Hares M.K., Marcelli G., Sirlantzis K. (2022). Performance of Deep Learning Models in Forecasting Gait Trajectories of Children with Neurological Disorders. Sensors.

[31] Lim J., Park S., Choi D., Bok K., Yoo J. (2022). Road Speed Prediction Scheme by Analyzing Road Environment Data. Sensors.

